# Supplementary material for: Implantation initiation of self-assembled embryo-like structures generated using three types of mouse blastocyst-derived stem cells
Source: Nat Commun. 2019 Jan 30;10:496. doi: 10.1038/s41467-019-08378-9 (PMC6353907; doi:10.1038/s41467-019-08378-9)
Supplement: Supplementary file 1 — Supplementary Information [file 41467_2019_8378_MOESM1_ESM.pdf]

Title: Implantation initiation of self-assembled embryo-like structure generated using three types of mouse blastocyst-derived stem cells

Shaopeng Zhang, et al.

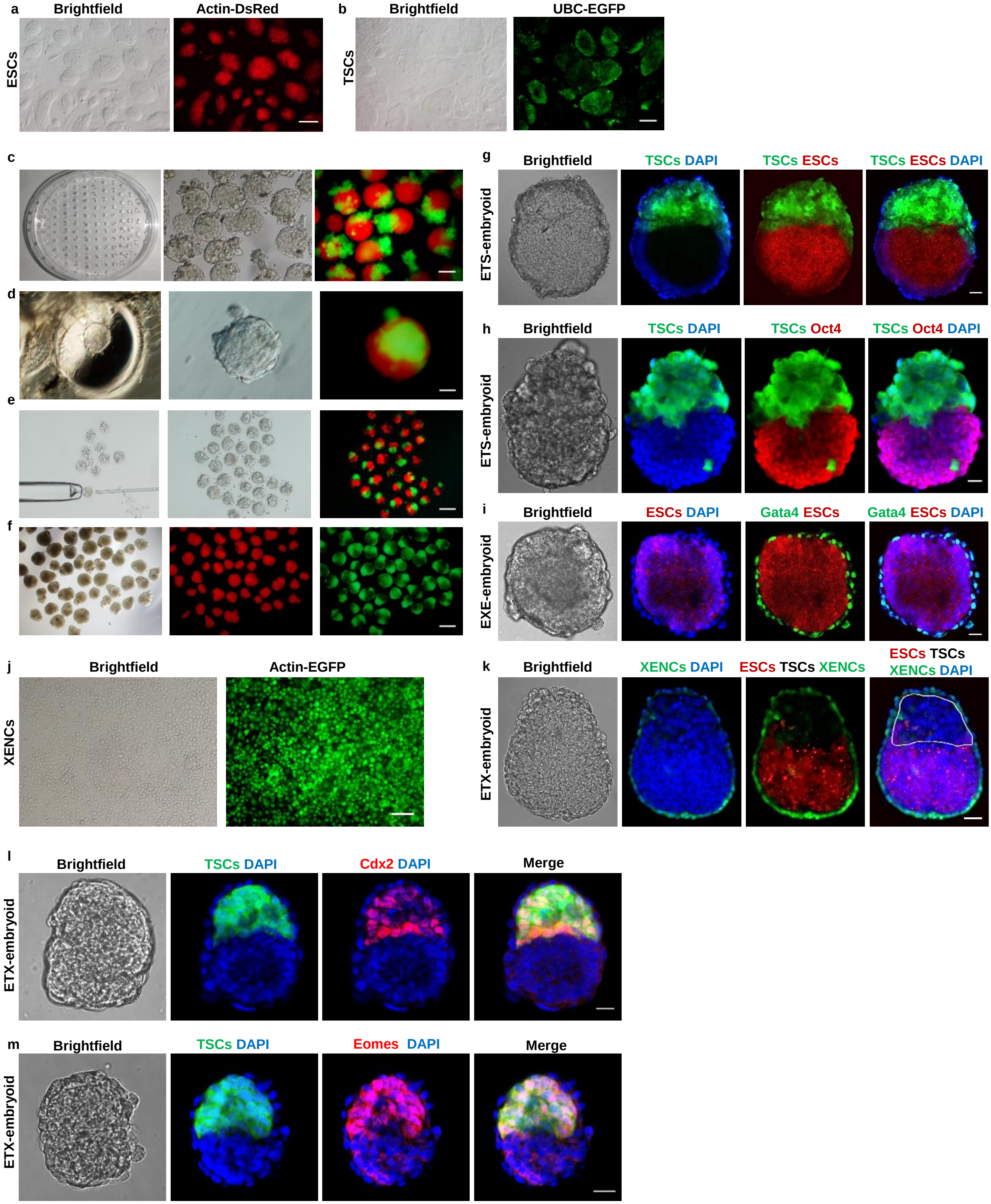

Supplementary Figure 1

Supplementary Figure 1 Optimization of the culture conditions of generation of the self-assembled embryos

a Morphology of G4 DsRed-ESCs. Scale bar, 100 µm.

b Morphology of EGFP-TSCs. Scale bar, 100 µm.

c-f The ETS-embryoid generation methods of hanging drop culture, microporous culture, microinjection method and non-adherent suspension shaking culture respectively. Left panels show how the ETS-embryoids were generated in these methods; middle panels show the population of the ETS-structures produced by the four methods; and right panels show the locations of the ESC- and TSC-compartments determined via DsRed and EGFP reporter proteins respectively. Scale bar, 100 µm (c, e); 50 µm (d); 200 µm (f).

g ETS-embryoids assembled from EGFP-TSCs and DsRed-ESCs after 84 hours of culture from **f**: DAPI, blue; TSC-derived tissues, green; ESC-derived tissues, red. n = 20 ETS-embryoids; two experiments. Scale bar, 50 µm.

h Oct4 staining to reveal the ESC compartment in ETS-embryoids assembled from EGFP-TSCs and wild-type ESCs. n = 20 ETS-embryoids; two experiments. Scale bar, 20 µm.

i Immunostaining of EXE-embryoids assembled from wild-type XENCs and G4 DsRed-ESCs after 60 hours of culture: the nuclei are stained with DAPI, blue; XENC-derived tissues are stained to reveal Gata4, green; ESC-derived tissues, red. n = 45 EXE-embryoids; three experiments. Scale bar, 20 µm.

j Morphology of EGFP-XENCs. Scale bar, 100 µm.

k ETX-embryoids assembled after 72 h of culture from wild-type TSCs, EGFP-XENCs and G4 DsRed-ESCs. The white line highlights TSC-derived tissues: DAPI, blue; XENC-derived tissues, green; and ESC-derived tissues, red. n = 24 ETX-embryoids; two experiments. Scale bar, 20 µm.

l-m Immunostaining of ETX-embryoids confirm the identity of TSC-derived tissues. TSCs, green; Cdx2, red, n=5 ETX-embryoids (l); Eomes, red (m); DAPI, blue. n = 5 ETX-embryoids (m). Scale bar, 20 µm.

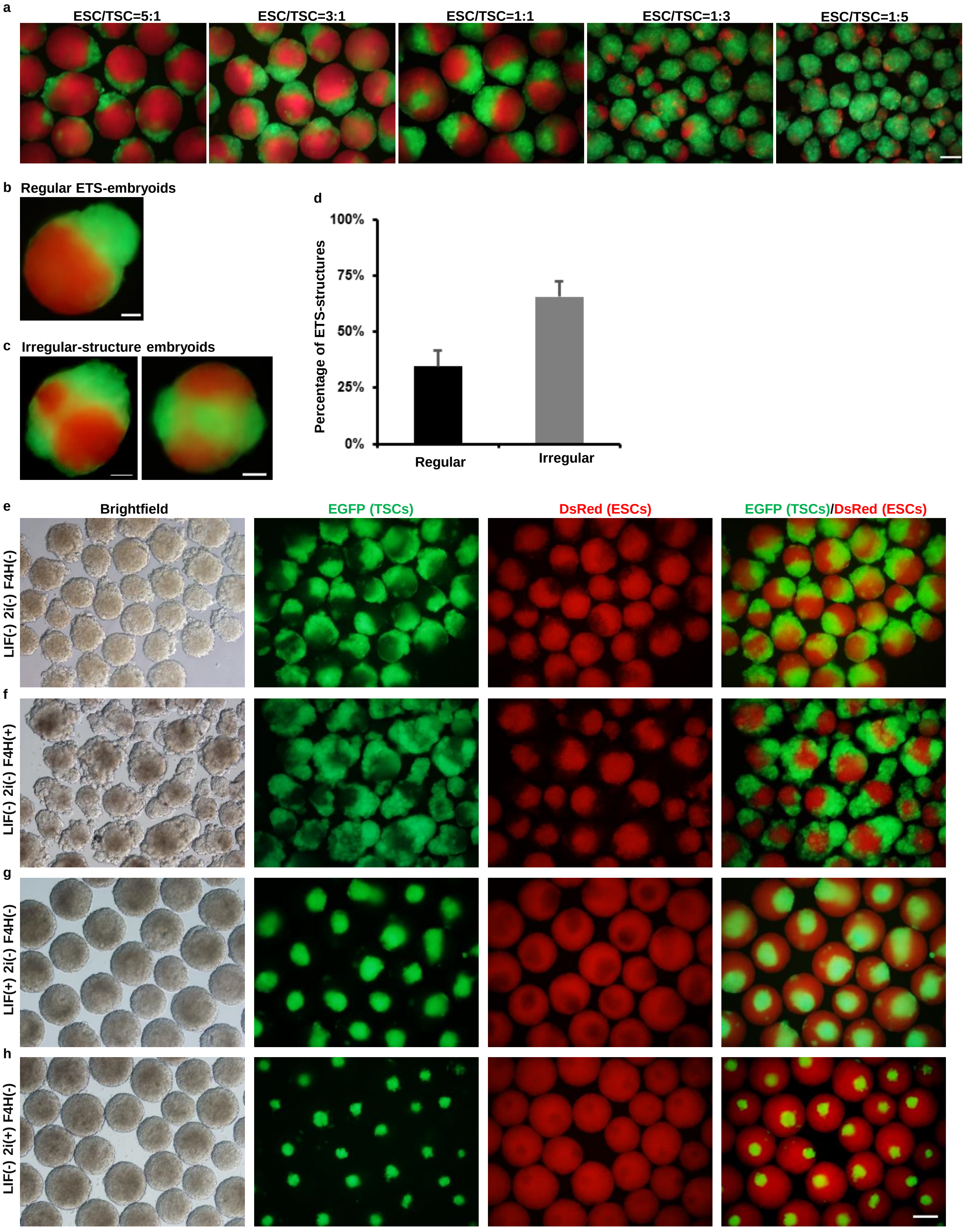

Supplementary Figure 2

Supplementary Figure 2 The formation of ETS-embryoids affected by initial cell ratios and medium compositions

a Initial cell ratios affect the sizes of the ESC- and TSC-compartments in ETS-embryoids. From left to right, the ESC/TSC ratios are 5:1 (5×10^5 ESCs and 1×10^5 TSCs in a 3.5-cm diameter dish), 3:1, 1:1, 1:3 and 1:5 consecutively. Scale bar, 200 µm.

b Representative ETS-embryoids with appropriate proportions and positions of ESC- and TSC-compartments. Scale bar, 50 µm.

c ESC- and TSC-derived irregular embryoids with TSC-ESC-TSC (left panel) and ESC-TSC-ESC (right panel) structures. Scale bar, 50 µm.

d Percentage of ETS-embryoids and irregular-structure embryoids. Five experiments. Columns are means $\pm$ s.d.

e-h 2i, LIF and FGF4 affect the formation of ETS-embryoids. **(e)** ETS-embryoids cultured with 1/2 ESC basal medium plus 1/2 TSC basal medium without 2i, LIF and FGF4 + Heparin (F4H), [LIF(-)2i(-)F4H(-)], show appropriate proportions and positions of ESC- and TSC-compartments. **(f)** ETS-embryoids cultured in 1/2 ESC basal medium plus 1/2 TSC basal medium with F4H, [LIF(-)2i(-)F4H(+)], show over-proliferation of TSC compartments. **(g)** ETS-embryoids cultured in 1/2 ESC basal medium plus 1/2 TSC basal medium with LIF, [LIF(+)2i(-)F4H(-)], show over-proliferation of ESC-compartments. **(h)** ETS-embryoids cultured in 1/2 ESC basal medium plus 1/2 TSC basal medium with 2i, [LIF(-)2i(+)F4H(-)], show over-proliferation of ESC compartments and inhibition of TSC proliferation. F4H: FGF4 25 ng/mL, heparin 1 µg/mL. Scale bar,100 µm.

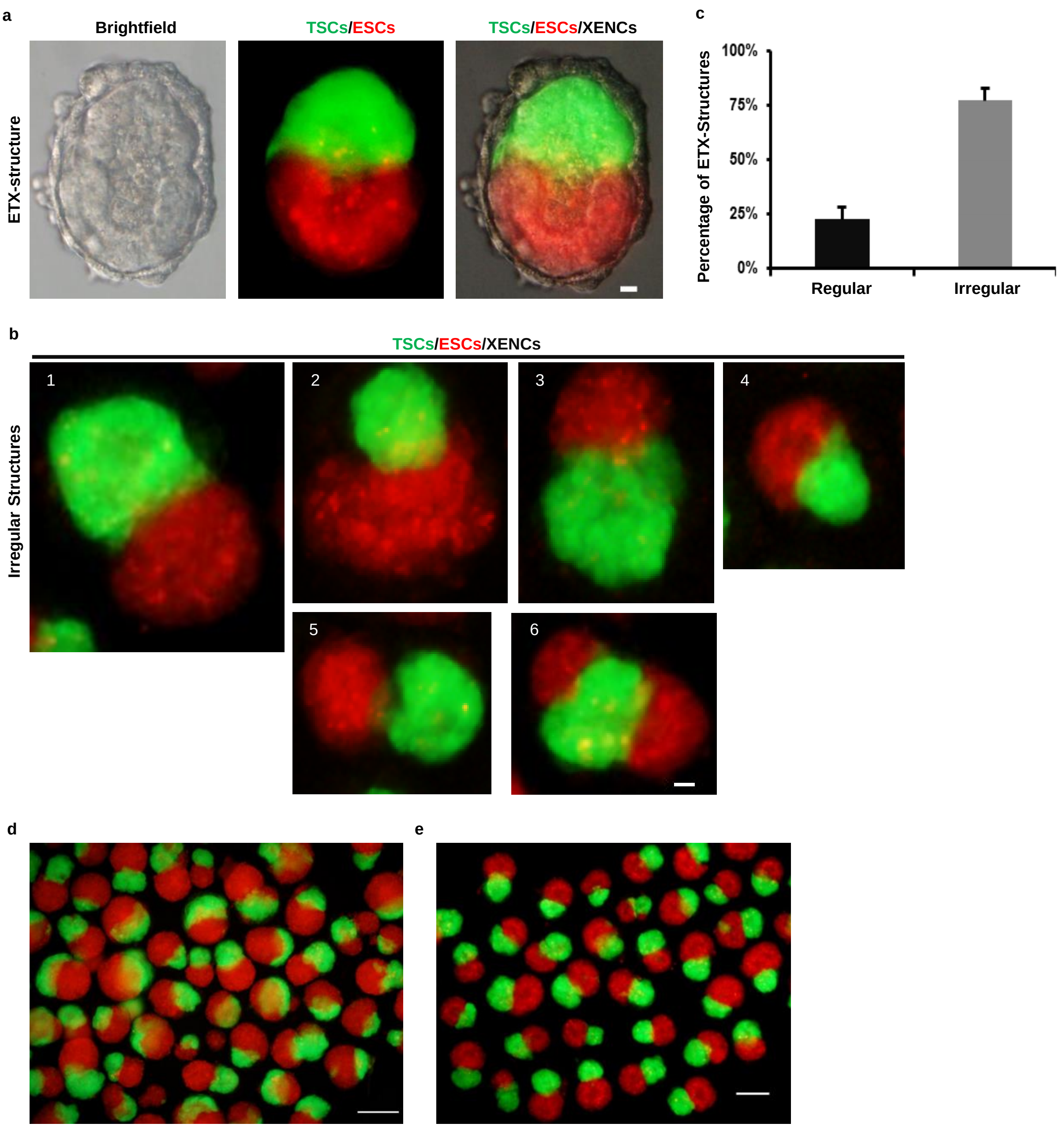

Supplementary Figure 3

Supplementary Figure 3 Evaluation and selection of ETX-embryoids

a ETX-embryoids assembled from EGFP-TSCs, wild-type XENCs and DsRed-ESCs, and cultured in LIF(-)2i(-)F4H(-) show appropriate proportions and positions of ESC- , TSC-compartments and XENC-derived tissues (localized at the outer layer of the ETX-embryoid), morphologically resembling egg cylinder stage of wild-type embryos.

b Representative irregular structures: Oversized whole ETX-embryoid (1); Oversized ESC-compartment (2); Oversized TSC-compartment (3); Undersized whole ETX-embryoid (4); ESC- and TSC-compartments not in close contact (5); Embryos with two ESC compartments (6). Scale bar, 20 µm.

c Percentages of ETX-embryoids with appropriate proportions and positions of ESC- and TSC-compartments and irregular structures. Eight experiments. Columns are means $\pm$ s.d.

d Representative population of ETX-embryoids before selection. Scale bar, 100 µm.

e Representative population of ETX-embryoids after selection. Scale bar, 100 µm.

All experiments were repeated at least three times with similar results.

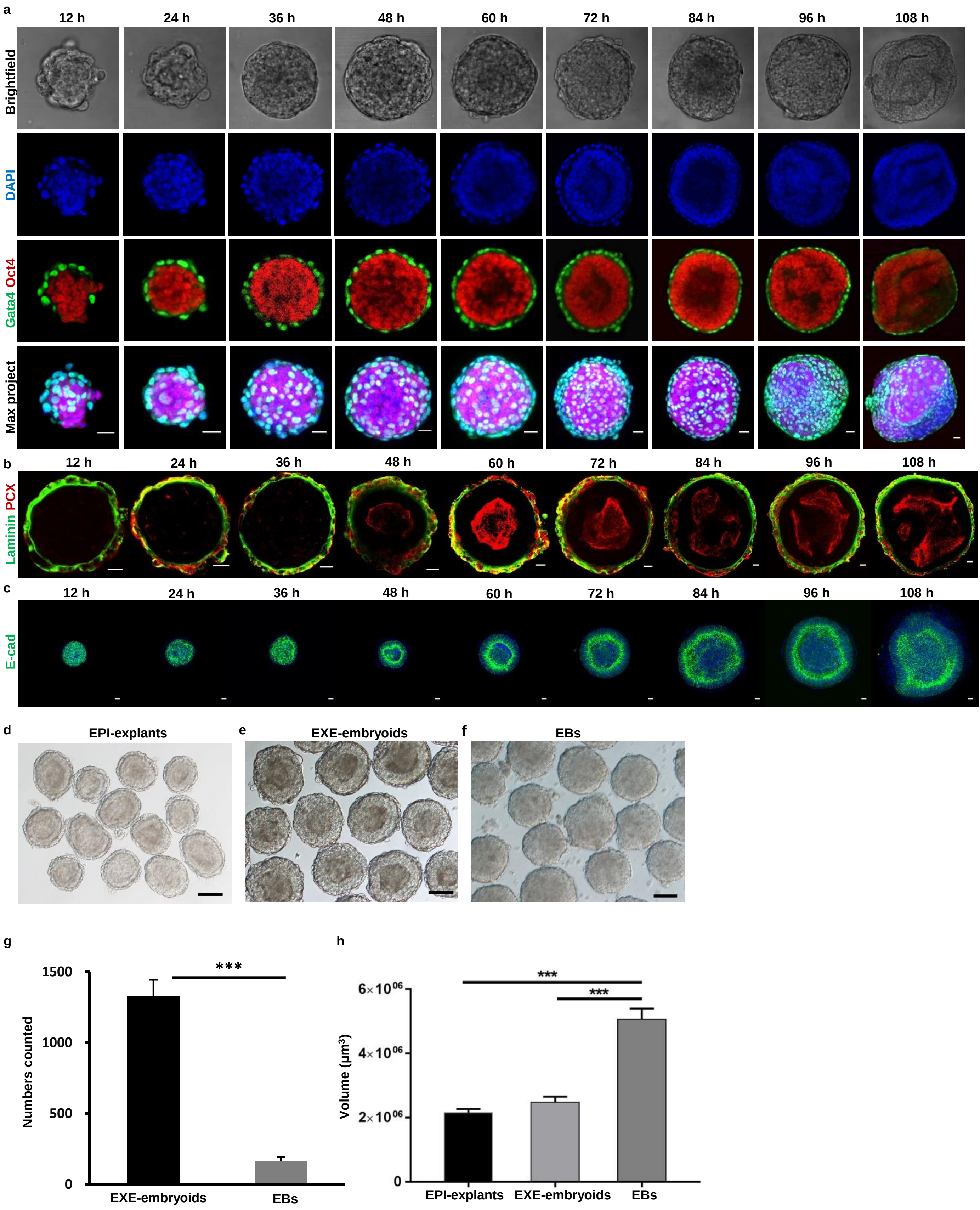

Supplementary Figure 4

Supplementary Figure 4 Morphogenetic development steps of EXE-embryoids

a Confocal images of representative EXE-embryoid immunostaining at multiple time points in which Gata4 (green) indicates XENC-derived tissues and Oct4 (red) indicates ESC-derived tissues. ESC compatments of EXE-embryoids surrouned by a XEN layer as shown in the 3D max. projection. For each time point, n = 15 EXE-embryoids, three experiments. Scale bar, 20µm.

b Immunostaining of Laminin (green) and PCX (red) indicates basal membrane niche and cavity formation and enlargement along with time in the EXE-embryoids. For each time point, n = 15 EXE-embryoids, three experiments. Scale bar, 20 µm.

c Immunostaining of E-cadherin (green) indicates cavity enlargement along with time in the EXE-embryoids at a series of time points. For each time point, n = 15 EXE-embryoids. Scale bar, 20 µm.

d-f Morphology of EPI-explants, EXE-embryoids and EBs respectively formed at 36 h, 60 h, 60 h in vitro cultured. Scale bar, 100 µm.

g Numbers of the EXE-embryoids and EBs formed at 60 h from the same initial cell numbers respectively. For EXE-embryoids, 1×10^5 ESCs and 1×10^5 XENCs; For EBs, 2×10^5 ESCs. Two-tailed Student's *t*-test, three experiments were performed. ***$P < 0.001$. Columns are means ± s.e.m.

h Volumes of the EPI-explants, EXE-embryoids and EBs cultured for 24 h, 60 h, 60 h respectively in vitro. The volume of EBs is significantly larger than EPI-explants and EXE-embryoids. Two-tailed Student's *t*-test, n = 11 EPI-explants, n = 24 EXE-embryoids and 40 Ebs. ***$P < 0.001$. Columns are means ± s.e.m.

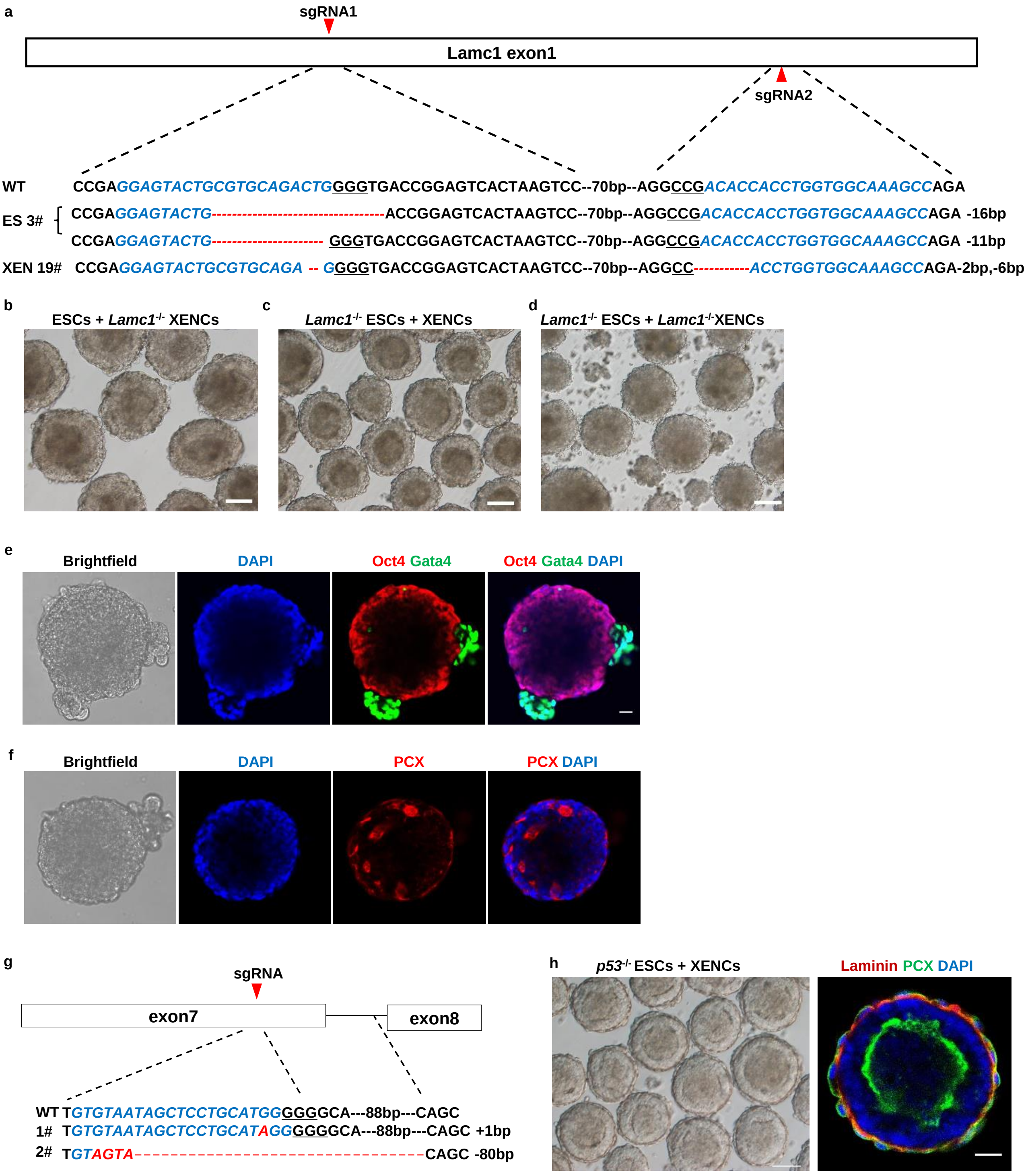

Supplementary Figure 5

Supplementary Figure 5 Roles of Lamc1 and p53 on generation and development of EXE-embryoids

a Schematic diagram of gene editing at the *Lamc1* loci. Wild-type sequences are shown at the top of the targeting sequence. Mutated sequences in ES cell line and XEN cell line are shown under the wild-type sequence. sgRNA1 and sgRNA2 are shown in blue. Protospacer adjacent moti (PAM) sequences are underlined. Insertions and deletions are highlighted in red. WT: wild-type; deletion "−"; insertion: "+".

b Representative EXE-embryoids assembled with wild-type ESCs and *Lamc1*$^{-/-}$ XENCs, showing regular morphology and cavity formation. n > 100 EXE-structures per group, three experiments. Scale bar, 100 µm.

c Representative EXE-embryoids assembled with *Lamc1*$^{-/-}$ESCs and wild-type XENCs, showing regular morphology and cavity formation. n > 100 EXE-structures per group, three experiments. Scale bar, 100 µm.

d Representative structures, assembled with *Lamc1*$^{-/-}$ ESCs and *Lamc1*$^{-/-}$ XENCs, show irregular morphology and failure of cavity formation. n > 50 structures, three experiments. Scale bar, 100 µm.

e Immunostaining of Oct4 (red) and Gata4 (green) indicates the locations of ESC- and XENC-derived tissues in the irregular structures which morphology is similar to EBs. XEN cells did not wrap ESC-compartments. n = 30 EXE-structures, two experiment. Scale bar, 20 µm.

f Immunostaining of PCX (red) indicates failure of basal membrane and cavity formation in the irregular structures. n = 30 EXE-structures, two experiment. Scale bar, 20 µm.

g Schematic diagram of gene targeting at the *p*53 gene loci. Wild-type sequences are shown at the top of the targeting sequence. sgRNA sequences are shown in blue. Protospacer adjacent moti (PAM) sequences are underlined. Insertions and deletions are highlighted in red. WT: wild-type; deletion "−"; insertion: "+".

h Left panel: EXE-embryoids assembled *p*53$^{-/-}$ G4 ESCs with WT XEN cells cultured for 72 hours in vitro. Scale bar, 100 µm; Right panel: Cavity of EXE-embryoids revealed by immunostaining, Laminin (red), PCX (green) and DAPI (blue). n = 20 EXE-embryoids, Two experiments were performed. Scale bar, 20 µm.

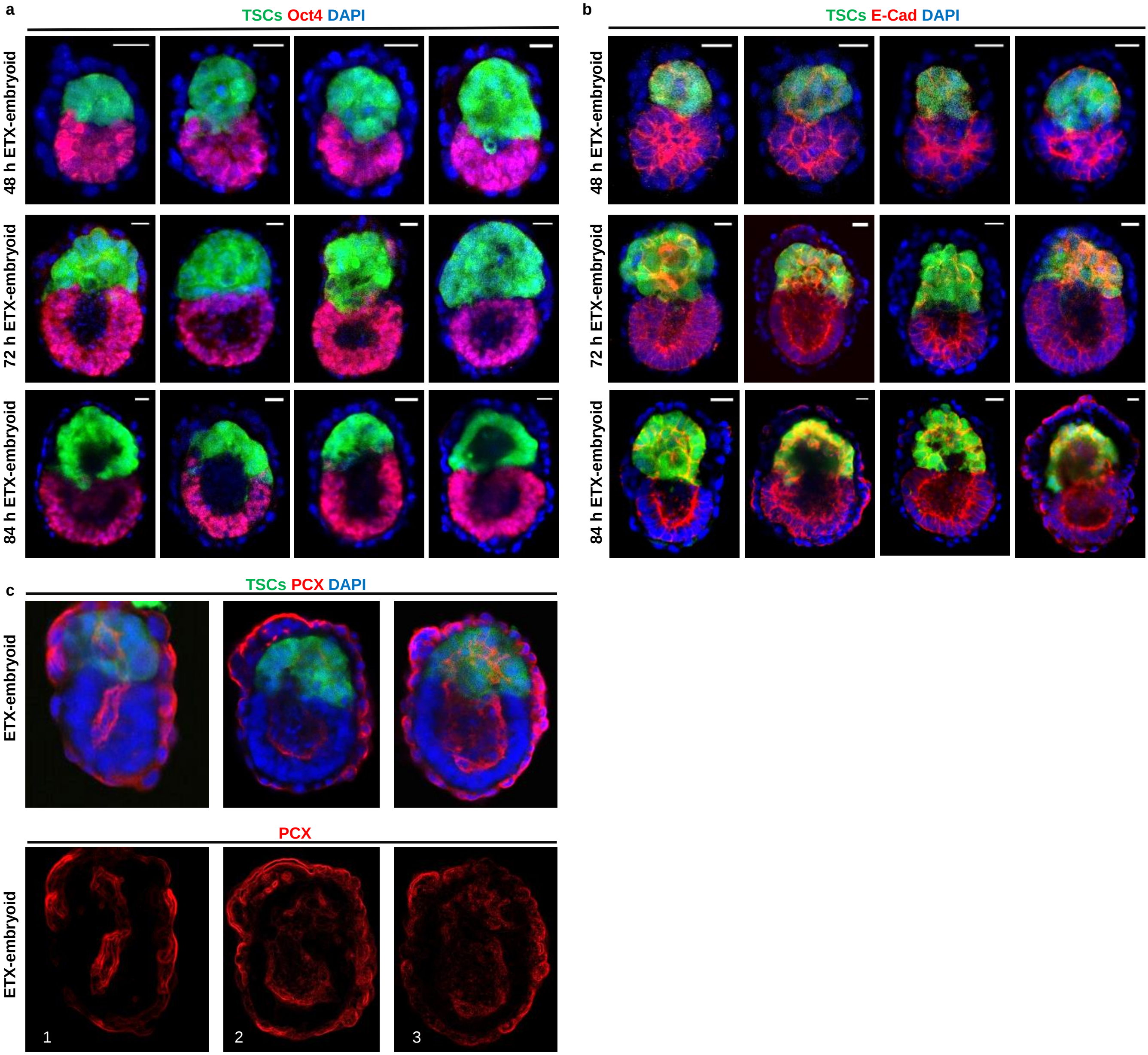

Supplementary Figure 6

Supplementary Figure 6 Morphogenetic steps leading to cavity formation of ETX-embryoids

a Representative ETX-embryoids at each time point of (48, 72 and 84 h) showing the localization of the ESC compartment (Oct4, red), the TSC compartment (EGFP-TSCs, green) and the XENC compartment (DAPI only, blue). n = 20 ETX-embryoids (48 h), n = 20 ETX-embryoids (72 h) and n = 20 ETX-embryoids (84 h); two experiments were performed.

b Representative ETX-embryoids at each time point of (48, 72 and 84 h), showing the progression of cavitation. UBC-EGFP TSCs indicate the TSC compartment (green); E-cadherin, red; DAPI, blue. E-cadherin accumulated along the apical sides of the cells in ESC compartments when a single lumen was observed at 48 h, while no accumulation was observed in the TSC-compartments at this stage. Accumulation of E-cadherin along the apical sides of the cells in TSC compartments occurred at 72 h, and the cavity in ESC compartments enlarged. By 84 hours, the cavities in the ESC- and TSC-compartments had combined into a common cavity. n = 27 ETX-embryoids (48 h), n = 22 ETX-embryoids (72 h), n = 22 ETX-embryoids (84 h); two experiments were performed.

c Analysis of the PCX distribution in the ESC- and TSC-compartments of ETX-embryoids as the cavities progressively enlarged using Find Edges Function by the Image J software. The cavities in both the ESC- and TSC-compartments enlarged and contacted to form an interface between them (panel 1). As the cavities grew, the interface extended and finally broke down, leading the two cavities to unify into a common cavity (panel 2 and 3). Scale bar, 20 μm.

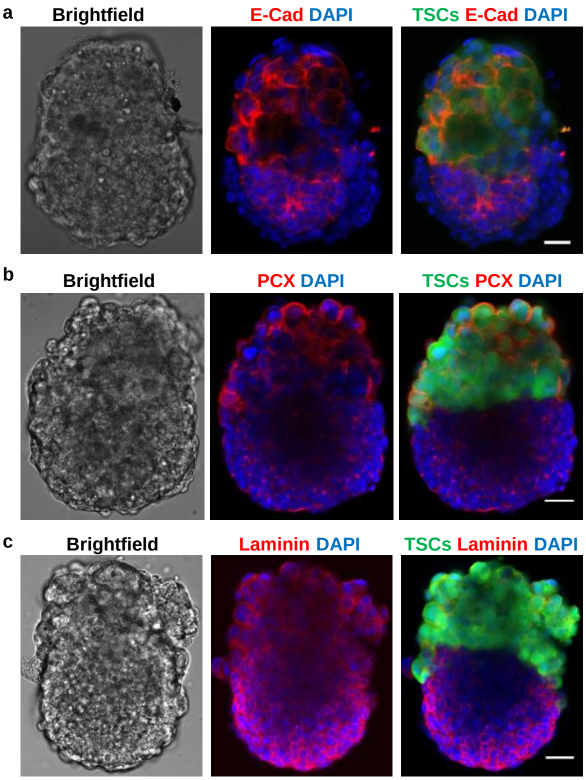

Extended Data Figure 7 | ETS-embryoids do not follow the spatial-temporal events of embryogenesis or acquire the characteristic architecture of a post-implantation embryo

a ETS-embryoids, in which EGFP-TSCs (green) indicate the TSC compartment, were stained to reveal E-Cadherin (red) and nuclei (DAPI, blue). n = 20 ETS-embryoids, three experiments. Scale bar, 20 µm.

b ETS-embryoids stained to reveal PCX (red), nuclei (DAPI, blue) and the TSC-compartment (green). n = 20 ETS-embryoids, three experiments. Scale bar, 20 µm.

c ETS-embryoids stained to reveal Laminin (red), nuclei (DAPI, blue), and the TSC-compartment (green). n = 20 ETS-embryoids, three experiments. Scale bar, 20 µm.

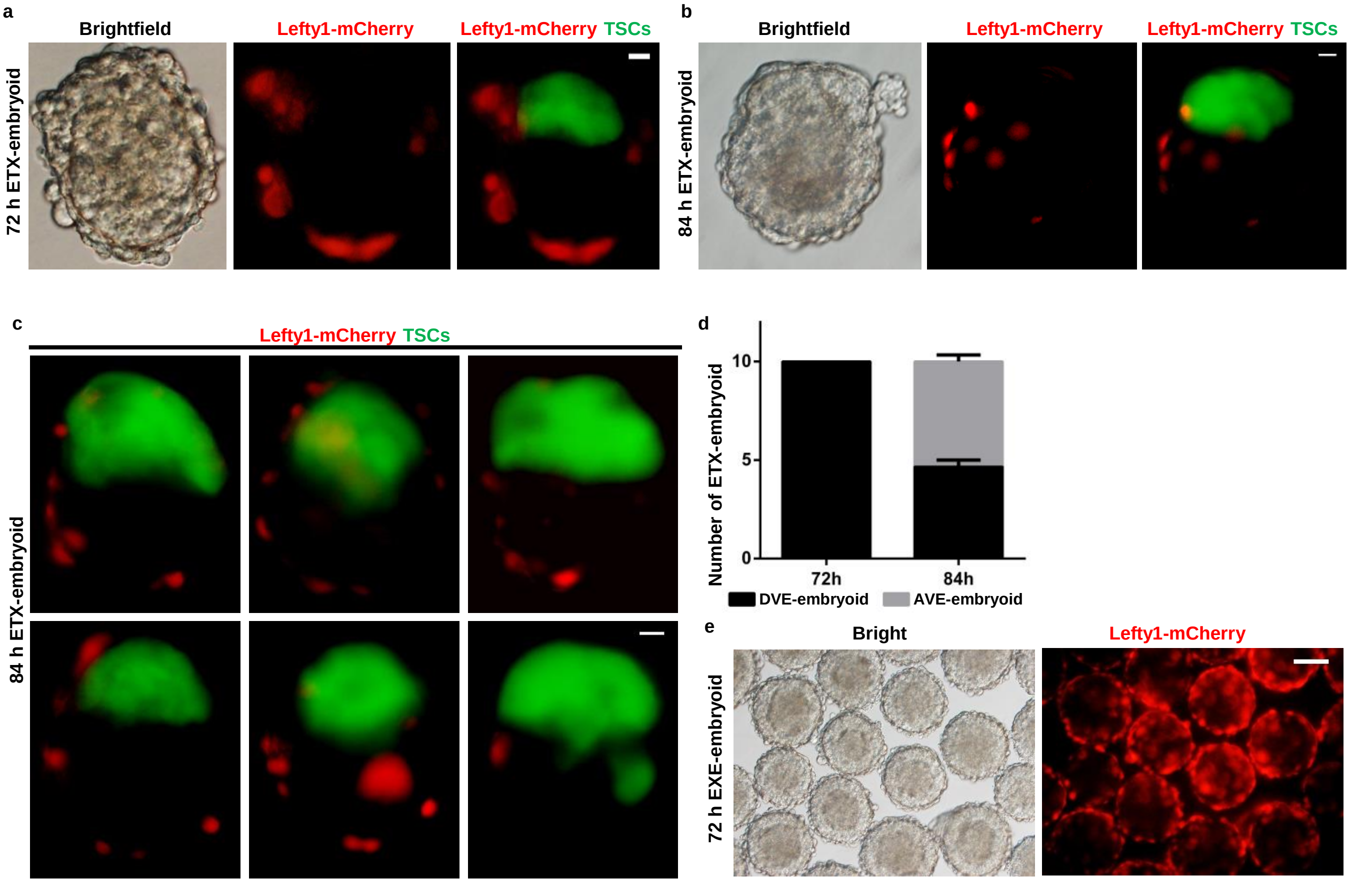

Supplementary Figure 8 DVE/AVE-like tissues formation in the ETX-embryoids

a Representative ETX-embryoids with *Lefty1*-mCherry positive XENC-derived AVE-like tissues at 72 h in vitro culture. n = 10 ETX-embryoids, three experiments. Scale bar, 20 µm.

b Representative ETX-embryoids with *Lefty1*-mCherry positive XENC-derived DVE-like tissues at 84 h in vitro culture. n = 10 ETX-embryoids, three experiments. Scale bar, 20 µm.

c Examples of ETX-embryoids generated from single experiment using *Lefty1*-mCherry XENCs to indicate DVE/AVE-like tissues formation. Scale bar, 20 µm.

d ETX-embryoids with DVE like features were selected at 72 h and cultured to 84 h. Quantification show the number of ETX-embryoids with DVE and AVE-like tissues at 72 h and 84h hours in vitro culture. n = 10 EXE-embryoids per group, three experiments.

e Representative EXE-embryoids with random distribution of *Lefty1*-mCherry positive cells at 72 h in vitro culture. n>30 EXE-embryoids, three experiments. Scale bar, 20 µm.

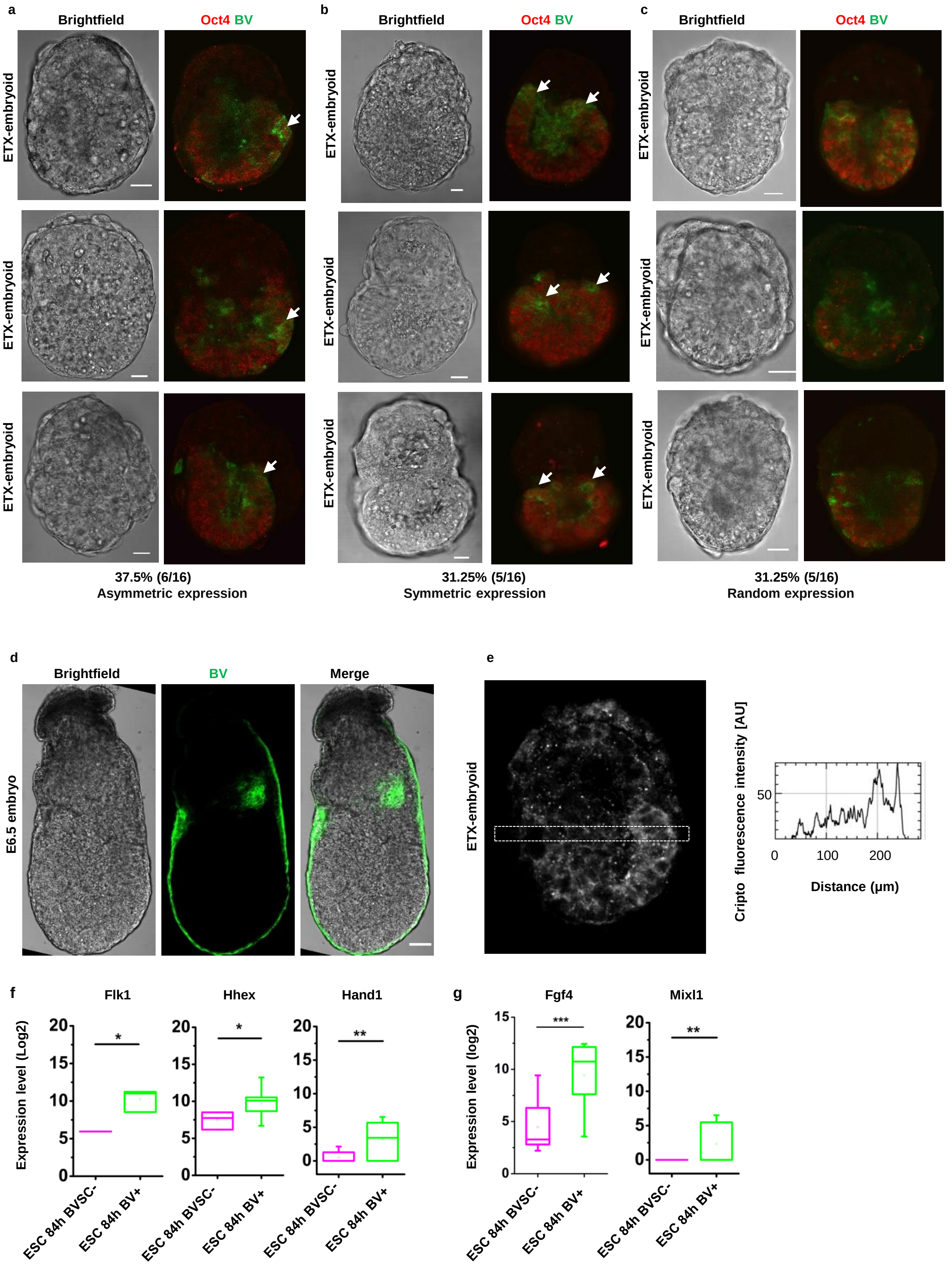

Supplementary Figure 9

Supplementary Figure 9 Three expression modes of Blimp1 in ETX-embryoids in which *Blimp1*-mVenus ESCs formed the ESC compartments

a Three representative ETX-embryoids showing asymmetric expression of the *Blimp1*-mVenus in ESC compartment close to the boundary between TSC-and ESC-compartments. n = 6 ETX-embryoids; two experiments. Scale bar, 20 µm.

b Three representative ETX-embryoids showing symmetric expression of the *Blimp1*-mVenus in ESC compartment close to the boundary between TSC-and ESC-compartments. White arrows indicated symmetric expression of the *Blimp1*-mVenus at the boundary between TSC-and ESC-compartments. n = 5 ETX-embryoids; two experiments. Scale bar, 20 µm.

c Three representative ETX-embryoids showing random expression of the *Blimp1*-mVenus in ESC-compartments of the ETX-embryoids. n = 5 ETX-embryoids; two experiments were performed. Scale bar, 20 µm.

d Distribution of BV positive cells in the BVSC E6.75 embryos. n = 10 embryos; two experiments. Scale bar, 20 µm.

e White box used to plot intensity profiles of Cripto fluorescence within ESC-compartment.

f-g Single-cell RT-qPCR analysis of the expression of mesoderm markers (*Flk1, Hhex* and *Hand1*) and the anterior EPI markers (*Fgf4*, *Mixl1*) in *Blimp1*-mVenus-positive (BP) and *Blimp1*-mVenus-negative (BN) cells from the ESC compartments of the ETX-embryoids. Box plots are generated as described in Figure 1h. Two-tailed Student's *t*-test, n = 11 single cells (84 h, BV-) and 16 single cells (84 h, BV+). *$P < 0.05$, **$P < 0.01$, ***$P < 0.001$.

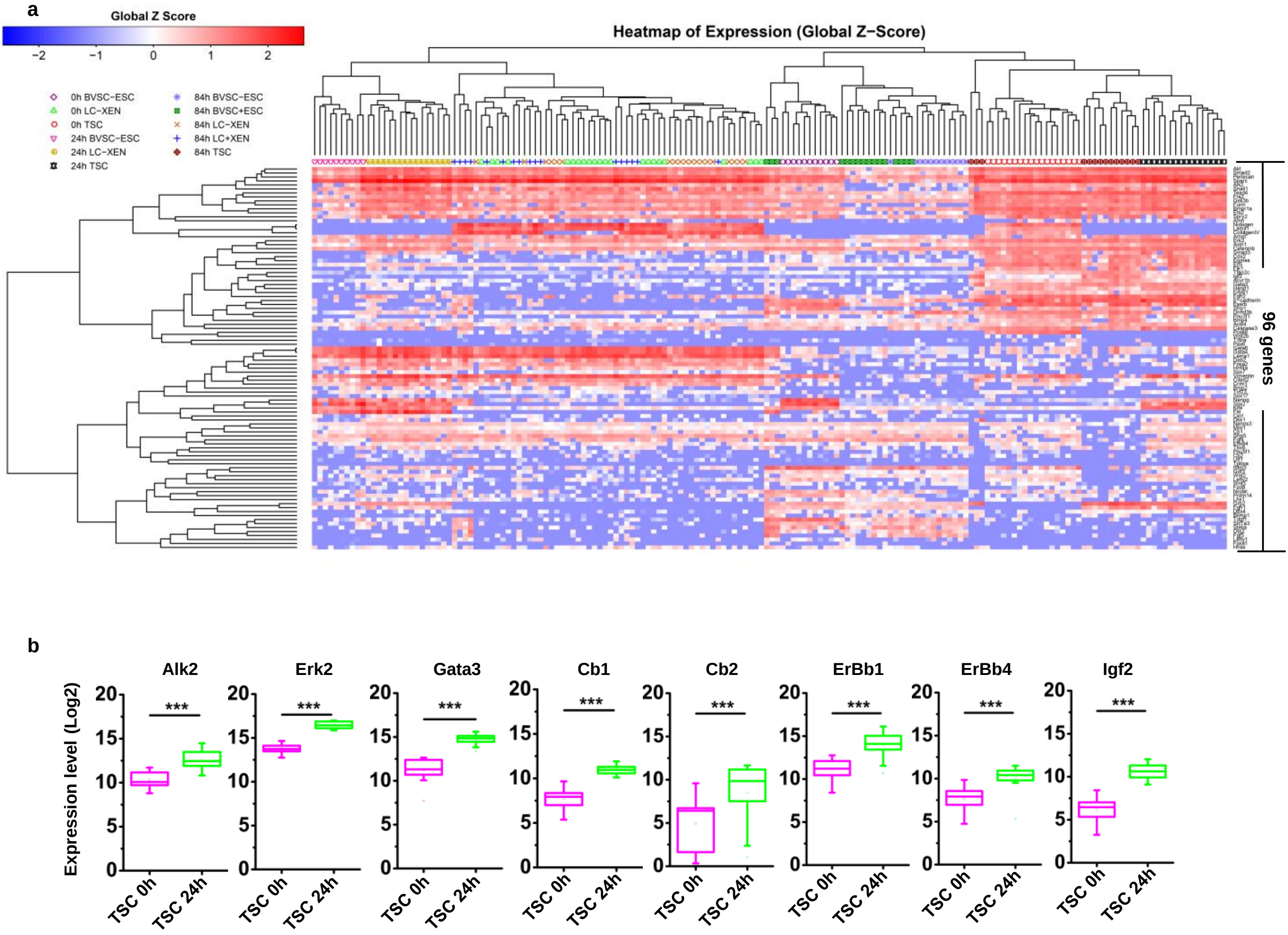

Supplementary Figure 10 ETX-embryoid developmental transition is defined by single cell gene expression profiles

a Hierarchical clustering of single cells isolated from ESC, TSC and XENC compartments at the initial stage (0 hour), 24 hours and 84 hours based on the expression levels of 96 genes.

b Box plots of expression levels of the markers related to implantation, such as *Alk2, Erk2, Gata3, Cb1, Cb2, ErBb1, ErBb4* and *Igf2*. Box plots are generated as described in Figure 1h. A background of Ct = 26 was used to obtain expression levels. n = 18 TSCs 0 h and n = 16 TSCs 24 h. ****P* < 0.001. Columns are means ± s.e.m.

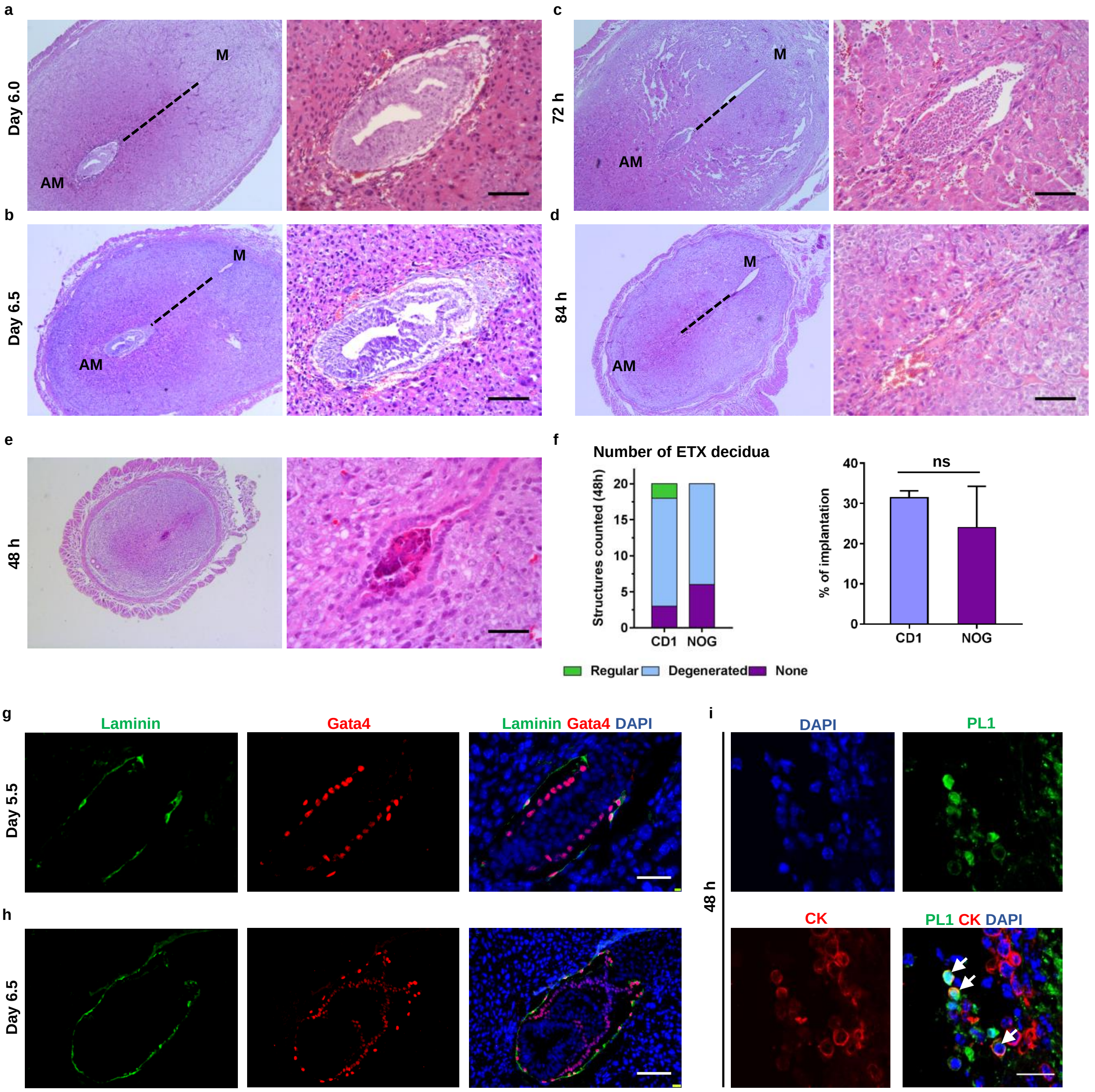

Supplementary Figure 11

Supplementary Figure 11 Degeneration of the ETX-embryoids after implantation

a-b Representative cross-section of E6.0 and E6.5 wild-type embryo implantation sites. The yellow dotted line shows the embryonic long axis. Right panel show the magnified embryonic image. AM, antimesometrial pole; M, mesometrial pole. n = 2 deciduas. Scale bar, 100 µm.

c-d Representative cross-section of a degenerated ETX-embryoid at 72 h and 84 h after transplantation. The yellow dotted line shows the embryonic long axis. Zoomed fields highlight the degenerated ETX-embryoid. AM, antimesometrial pole; M, mesometrial pole. n = 6 deciduas. Scale bar, 100 µm.

e Representative cross-section of a degenerated ETX-embryoid implantation site 48 hours after transplantation into NOG recipient mouse. n = 6 deciduas. Scale bar, 50 µm.

f Comparison of the ETX-embryoid development potential in CD1 and NOG recipients. Left panel show the number of the regular and degenerated structures in the deciduae of transferred ETX-embryoids. n = 20 deciduas. Right panel show the percentage of the implantation of the transferred ETX-embryoids in CD1 and NOG recipients. Two-tailed Student's *t*-test. ns, not significant. Columns are means $\pm$ s.e.m. Five experiments.

g-h Immunostaining of Laminin (green) and Gata4 (red) in day 5.5 and day 6.5 uterine deciduae that indicate the basal membrane and the tissues derived from PE. n = 10 deciduas for each group, two experiments. Scale bar, 50 µm (g) and 100 µm (h).

i Co-immunostaining of PL1 (green) and CK (red) in sections of implantation sites on 48h after ETX-embryoid transplantation. Signals for PL1 and CK show trophoblast cells and the uterine luminal epithelium (LE) interaction. White arrows point toward trophoblast cells (green) actively engulf proximate LE cells (red). Scale bar, 20 µm.

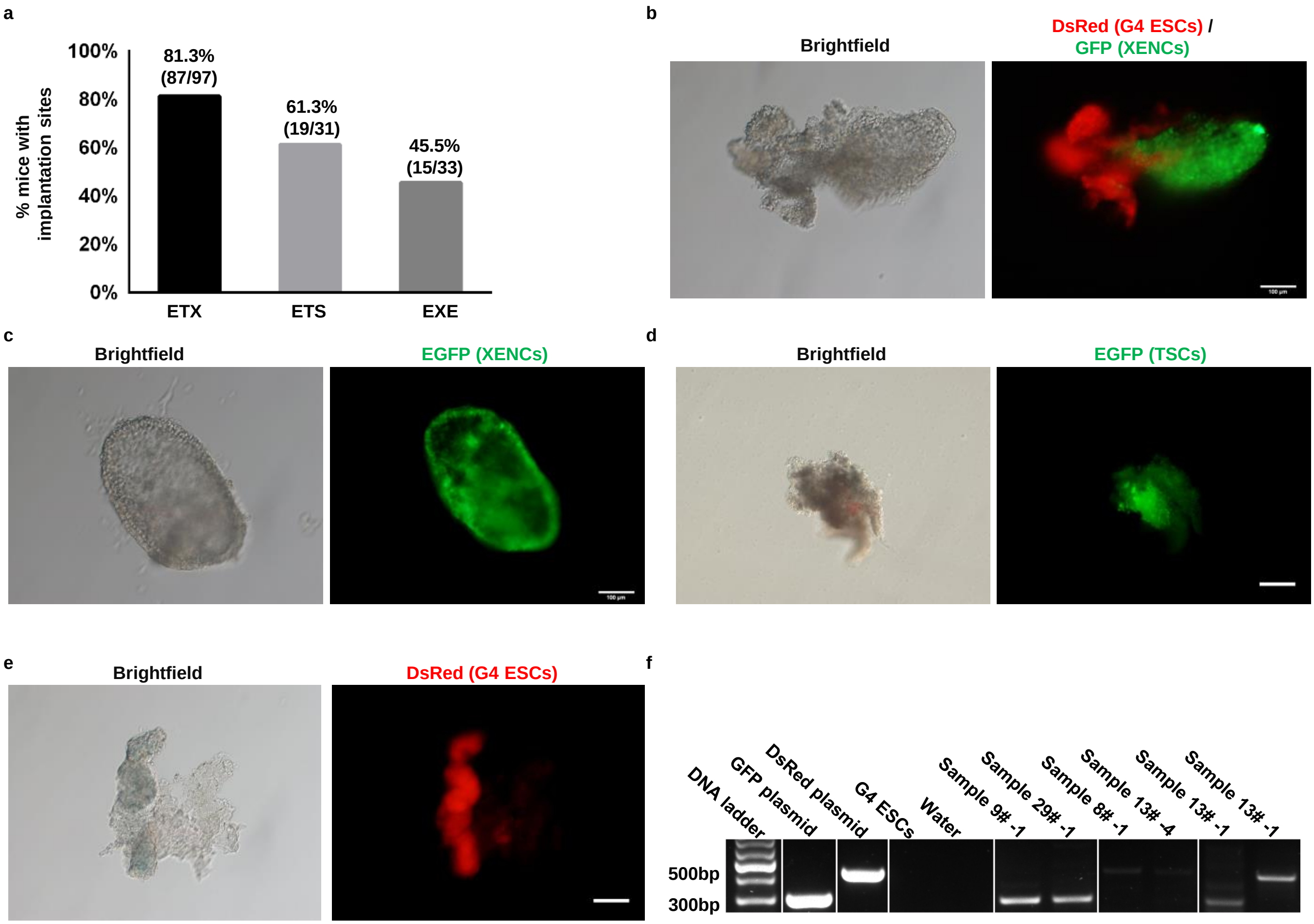

g

Number of pregnant mice	DsRed (G4 ESCs) / EGFP (TSCs)	DsRed (G4 ESCs) / EGFP (XENCs)	EGFP (XENCs) only	EGFP (TSCs) only	DsRed (G4 ESCs) only
9	2	1	2	1	11

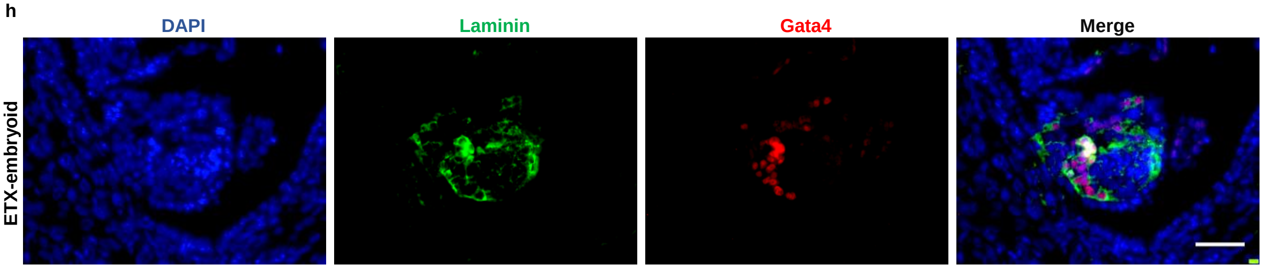

Supplementary Figure 12

Supplementary Figure 12 Identification of the transferred ETX-embryoids in the decidua tissues

a The number of mice with implantation sites per total number of mice examined after transplantation of ETX-, ETS- and EXE-embryoids. The molecules and denominators in the fractions and in brackets indicate the number of mice containing implantation sites and the numbers of recipient mice respectively.

b-e Degenerated ETX-embryoids isolated from the deciduae. Representative embryo-like structure showing the disorder of ESC- and XENC-compartments (**b**); Representative structure derived from only XENCs (**c**); Representative structure derived from only TSCs (**d**); Representative structure derived from only ESCs (**e**). Scale bar, 100 µm.

f PCR analysis confirmed the genomic integration of GFP and DsRed in the isolated tissues derived from the related reporter stem cells. Sample 13#-1 from **b**, Sample 9#-1 from **c**, Sample 29#-1 from **d**, Sample 8#-1 and 13#-4 from **e**.

g Statistical analysis of the tissues isolated from implantation sites from **b-e**.

h Immunostaining analysis of Gata4 (red) and Laminin (green) in a degenerated ETX-embryoids implantation site at 72 h after transplantation. Scale bar, 50 µm.
