# Supplementary material for: Implantation initiation of self-assembled embryo-like structures generated using three types of mouse blastocyst-derived stem cells
Source: Nat Commun. 2019 Jan 30;10:496. doi: 10.1038/s41467-019-08378-9 (PMC6353907; doi:10.1038/s41467-019-08378-9)
Supplement: Supplementary file 5 — Description of Additional Supplementary Files [file 41467_2019_8378_MOESM5_ESM.docx]

**Title: Source Data**: Raw data for statistical analysis

**Description:** Summarized the qualifications of the data (Fig. 1i, 2h, 2i, 3k, 4c, 4f, 5e and 5f; Supplementary Fig. 2d, 3c, 4g, 4h and 8d) and the embryo transplantation data (Fig. 7b, 7c, 7m and 7n; Supplementary Fig. 11f, 12a, 12f).

**Title: Supplementary Data1:
Description:** Primers and raw data for single cell gene expression analysis

Summarized the raw data of the single-cell quantitative PCR experiments (Fig. 1h, 5g, 5i, 5j, 6d-g and 6i; Supplementary Fig. 9f, 9g, 10a and 10b) and the list of the gene with corresponding primer sequences.
